# Electro-Hemostosis in Operative Surgery

**Published:** 1900-03

**Authors:** 


ELECTRO-HEMOSTOSIS IN OPERATIVE SURGERY.—By Alexander J. C. Skene, M. D., LL. D., Professor of Gynecology in the Long Island College Hospital, New York, etc. Eighty illustrations and two plates. 173 pages. New York: D. Appleton & Company. 1899.

The author intends that this work should supplement the third edition of his work on diseases of women. In the introduction cat gut and silk ligatures are discussed in comparison with electro-hemostosis. From the author's standpoint of view there is no ligature equal to the electric cautery for staunching blood in vessels of any size. He has special instruments made by Mr. Pignolet, an electrician, and demonstrates the superiority of the method in all kinds of abdominal operations. The author employs and recommends the electro-cautery in the treatment of a great many conditions—pelvic abscesses and diseases of the vulva and vagina, for extirpating the mammary and other glands, tumors in the urethra and elsewhere; hemorrhoids, various neoplasms of the skin and mucous membranes.

It is a book that can be read with profit, for it is not along beaten paths. T. J. B.

